# The effect of Thyroid Autoimmunity on Dyslipidemia in patients with Euthyroid Hashimoto Thyroiditis

**DOI:** 10.12669/pjms.37.5.3883

**Published:** 2021

**Authors:** Hasret Cengiz, Taner Demirci, Ceyhun Varim, Ali Tamer

**Affiliations:** 1 Hasret Cengiz, Department of Endocrinology and Metabolism, Sakarya University Medicine Faculty, Sakarya, Turkey; 2 Taner Demirci, Assistant Professor, Department of Endocrinology and Metabolism, Sakarya University Medicine Faculty, Sakarya, Turkey; 3 Ceyhun Varim, Associate Professor, Department of Internal Medicine, Sakarya University Medicine Faculty, Sakarya, Turkey; 4 Prof. Ali Tamer, Department of Internal Medicine, Sakarya University Medicine Faculty, Sakarya, Turkey

**Keywords:** Dyslipidemia, Hashimoto thyroiditis, Thyroid autoimmunity

## Abstract

**Background & Objective:**

The relationship between thyroid disorders and dyslipidemia has now been clearly demonstrated, but the relationship between thyroid autoimmunity and dyslipidemia in euthyroid patients is still controversial. Our aim in this study was to determine whether there is a risk of dyslipidemia in our patients with euthyroid hashimoto thyroiditis.

**Methods:**

Fifty-five autoantibody positive hashimoto patients and 55 antibody negative control groups who came to Sakarya University Medicine Faculty Endocrinology Outpatient Clinics between January 2018 and November 2019 were included in our case control study. The groups were similar in terms of age, cardiovascular risk factors and BMI. Both groups were compared according to the lipid profile results.

**Results:**

All type of lipids; including LDL (p = 0.008), HDL (p = 0.041), triglyceride (p = 0.045) and total cholesterol (p = 0.002), were higher in the patient group, and these differences were statistically significant. Anti-TPO and Anti-TG antibody titers and lipid levels were evaluated by separate correlation analysis. There was a significant positive correlation between Anti-TPO and LDL (r = 0.331, p <0.001), triglyceride (r = 0.267, p = 0.005) and total cholesterol (r = 0.316, p = 0.001), however no significant correlation was observed between Anti-TPO and HDL. Similarly, there was a significant positive correlation between Anti-TG and LDL (r = 0.318, p = 0.001), triglyceride (r = 0.218, p = 0.022), and total cholesterol (r = 0.301, p = 0.001), but HDL correlation relationship was not detected.

**Conclusion:**

The relationship between thyroid autoimmunity and dyslipidemia has been demonstrated in our study even in the euthyroid phase. Whether antibody positive patients should be followed more closely for dyslipidemia and cardiovascular events is still controversial. This question will be answered with larger randomized controlled trials.

## INTRODUCTION

Chronic lymphocytic thyroiditis (Hashimoto thyroiditis); is the most common organ-specific autoimmune disease and the most common cause of thyroid dysfunction in iodine sufficient areas.^1^ Now, the disease, which is seen in almost 10% of the population more frequently in women than in men.^2^ Autoimmune thyroiditis causes a progressive thyroid dysfunction as a result of infiltration with inflammatory cells and fibrosis of the thyroid tissue in the chronic period. High levels of Anti-TPO and Anti-Tg antibodies are the most common laboratory finding of autoimmune thyroid disease.^3^,^4^

Dyslipidemia is the presence of high quality and quantity lipid and lipid metabolites in the circulation due to congenital or acquired reasons. Initially it is not clinically symptomatic usually, it is detected by chance in laboratory tests or it is noticed by vascular complications as a result of atherosclerosis secondary to dyslipidemia. Early detection of dyslipidemia can help prevent atherosclerosis thus preventing these unwanted events.

The main lipid forms in the blood that are routinely examined are Total cholesterol (TC), Low-density Lipoprotein (LDL), High Density Lipoprotein (HDL) and Triglyceride (TG). Total cholesterol and LDL are known primarily as atherogenic lipids. In addition, Triglyceride is a component of the metabolic syndrome and indirectly contributes to the pathogenesis of atherosclerosis. HDL cholesterol is the cholesterol known as good cholesterol, which collects cholesterol esters from peripheral tissues and atherogenic plaque and takes it to the liver. In addition, according to the new studies, HDL cholesterol provides resistance against atherosclerosis with many anti-inflammatory and anti-oxidant effects.^5^,^6^

Thyroid hormones affect the activity of many key enzymes in lipid metabolism, so lipoprotein composition and transport are severely impaired in thyroid diseases. Obvious hypothyroidism causes hypercholesterolemia and elevated LDL levels. The cause of hyperlipidemia in hypothyroidism is decreased LDL receptor count and decreased LDL excretion in the liver. In severe hypothyroidism, HDL is often slightly increased, but sometimes normal or slightly decreased. This is due to changes in hepatic lipase (HL) and cholesterol ester transfer protein (CETP) activity. The net result is a microenvironment where atherogenic lipids predominate. In addition, oxidation of lipids increases in hypothyroidism and oxidative stress load occurs.^7^-^9^

Subclinical hypothyroidism is a more common entity than overt hypothyroidism. Lipid changes are generally similar to overt hypothyroidism in subclinical hypothyroidism.^10^,^11^ The relationship of overt and subclinical hypothyroidism to dyslipidemia and atherosclerosis has now been more clearly demonstrated, while the relationship of thyroid autoimmunity to dyslipidemia has been less proven. Our aim in this study was to determine whether there is a risk of dyslipidemia in our patients with euthyroid hashimoto thyroiditis.

## METHODS

This study has been approved by the local ethics committee of Sakarya University (71522473/050.01.04/458. 31/08/2020). It was conducted in accordance with the ethical standards laid down in the Declaration of Helsinki (as revised in 2013).

Fifyfive euthtyroid autoimmune thyroiditis patients and 55 age matched healthy controls over the age of 18 who came to our Outpatient Clinics between January 2018 and November 2019 were included in the study. Diagnosis of autoimmune thyroiditis was made by determining thyroid autoantibody (Anti-TPO, Anti-Tg) positivity and detecting diffuse heterogeneity compatible with chronic thyroiditis on ultrasound. Thyroid Ultrasounds were performed by the same author with B Mode High Resolution USG device Logic 9, General Electric USA^®^. After measuring the height of the patients, BMI was calculated by measuring automatically with the device of TANİTA BF-300 as kg/m^2^.

Patients with known thyroid disease and using thyroid medication, with cardiovascular disease or congestive heart failure, smokers, using drugs known to affect lipid metabolism (statins, beta blockers, steroids, oral contraceptives, antipsychotics, etc.), pregnant women, with chronic kidney failure and chronic liver disease and diabetes mellitus were excluded from the study. Informed consent form was obtained from the patients and control group for the study.

### Laboratory

Fasting blood glucose was studied with Beckman Coulter AU5800 CA USA^®^ device with the suitable reagents and kits by glucose oxidase method. Biochemical parameters (Alanine Aminotranferase and Creatinine) were also studied by the same device.Insulin resistance was calculated using the Homeostatic Model Assesment of Insulin Resistance (HOMA-IR) method.^12^

Serum free T4, TSH, Anti-TPO and Anti-TG levels were measured by the chemiluminescent microparticle immunological method in the Abbott Architect I 2000 SR^®^ device with appropriate commercial reagents and kits.Total cholesterol and Triglyceride levels were measured by enzymatic colorimetric (glycerol oxidase/peroxidase) method. HDL cholesterol was measured after precipitation with phosphotungstate. Beckman coulter AU5800 CA USA^®^. LDL cholesterol was calculated with the friedewald formula.^13^

The cut off value for Anti-TPO positivity was 35 IU /ml and 40 IU / ml for Anti-Tg, the normal range for the TSH level was taken as 9.1-19.9pmol / L for 0.3-4.6 µU / ml ft4 in accordance with laboratory calibration guidelines.

### Statistical analysis

Data analysis was performed by using SPSS-22 for Windows (Statistical Package for Social Science, SPSS Inc. Chicago IL, USA^®^). The variables were investigated using visual (histograms, probability plot) and analytical methods (Kolmogorov-Simirnov/Shapiro-Wilk’s test) to determine whether or not they are normally distributed. We performed analyses to describe and summarize the distributions of variables. The continuous variables were expressed as mean and standard deviation or as median and interquartile range, depending on the normality of their distribution. The differences between qualitative/categorical variables such as gender distribution between groups were compared with the chi-square test since the values observed in the cells provided assumptions. The Mann–Whitney test was used to compare the variables that were not normally distributed. On the other hand, Student’s t test was used to compare the variables with normal distribution. While investigating the associations between non-normally distributed variables, the correlation coefficients and their significance were calculated using Spearman test. The statistically significant two tailed p-value was considered as <0.05.

## RESULTS

The study included 110 participants, including 55 euthyroid autoimmune thyroiditis patients with positive thyroid autoantibody and 55 autoantibody negative healthy control groups. The mean age was calculated as 28.8 ± 8.3 in the patient group and 27.5 ± 7.8 in the control group (p = 0.400). The gender distribution was the same in both groups and the female / male ratio was 51/4. The mean TSH in the patient and control groups was 1.86 ± 1.05 and 1.42 ± 0.87, respectively, and the difference was statistically significant (p = 0.019). Other descriptive statistics and comparison of these groups are summarized in Table-I.

**Table-I:** Comparison of descriptive statistics between the groups.

	Patients* (n=55)	Controls* (n=55)	P-value
Age (years)	28.8±8.3	27.5±7.8	0.400
Gender [W/M] (percentage)	51/4 (92.7/7.3)	51/4 (92.7/7.3)	NS
BMI (kg/m^2^)	22.89 ±3.78	23.60±3.54	0.309
TSH (mIU/L)	1.86±1.05	1.42 ±0.87	0.019
fT4 (pmol/L)	14.55±1.76	15.28±1.81	0.035
Anti-TPO (IU/mL)	100.00 (43.07-336-78)	0.07 (0.01-0.20)	<0.001
Anti-TG (IU/mL)	47.41 (11.16-155.95)	0.95 (0.63-1.80)	<0.001
ALT (IU/L)	14.0 (11.0-18.0)	13.0 (10.0-17.0)	0.193
Cr (mg/dL)	0.70 (0.60-0.80)	0.70 (0.60-0.80)	0.421
Fasting blood glucose (mg/dL)	92.4±8.6	87.8±8.6	0.006
HOMA-IR	1.65 (1.21-2.22)	1.24 (0.86-1.78)	0.011

**Abbreviations:** BMI; body mass index, TSH; Thyroid-stimulating hormone, Anti-TPO; anti-thyroid peroxidase, Anti-TG; anti-thyroglobulin, ALT; Alanine transaminase, Cr; creatinine, HOMA-IR; homeostatic model assessment, NS; non significant.

* Descriptive results for continuous variables were expressed as mean and standard deviation or as median and interquartile range, depending on the normality of their distribution.

Both groups were compared according to the lipid profile results. All results, including LDL (p = 0.008), HDL (p = 0.041), TG (p = 0.045) and TC (p = 0.002), were higher in the patient group, and these differences were statistically significant (Table-II). Anti-TPO and Anti-TG antibody titers and lipid levels were evaluated by separate correlation analysis. There was a significant positive correlation between Anti-TPO and LDL (r = 0.331, p <0.001), TG (r = 0.267, p = 0.005) and TC (r = 0.316, p = 0.001), however no significant correlation was observed between Anti-TPO and HDL. Similarly, there was a significant positive correlation between Anti-TG and LDL (r = 0.318, p = 0.001), TG (r = 0.218, p = 0.022), and TC (r = 0.301, p = 0.001), but HDL correlation relationship was not detected.

**Table-II:** Comparison of lipid profiles between the groups.

	Patients*(n=55)	Controls* (n=55)	P-value
Low-density lipoprotein (LDL) (mg/dL)	105.24±28.44	92.07±22.13	0.008
High-density lipoprotein (HDL) (mg/dL)	59.47±14.97	54.33±10.74	0.041
Triglycerides (mg/dL)	84.0 (57.0-114.0)	72.0 (55.0-88.0)	0.045
Total cholesterol (mg/dL)	180.58±30.92	163.20±26.33	0.002

## DISCUSSION

In our current study, markedly increased TC, TG, LDL and HDL levels were found in euthyroid patients with positive autoantibody compared to the control group. In addition, fasting blood glucose and HOMA-IR levels were also significantly higher than the control group.

Thyroid hormones are involved in the activity of many important enzymes related to lipid metabolism. HMG-COA reductase activity, which is the key enzyme in cholesterol synthesis, increases with thyroid hormones. An increase in cholesterol synthesis is seen in hyperthyroidism. However, the net picture reflected in the laboratory is a decrease in atherogenic lipid levels such as TC and LDL cholesterol. This is due to the increase in hepatic LDL receptors and the net excretion of LDL. In hypothyroidism, on the contrary, excretion will decrease and atherogenic lipids will increase. Thyroid hormones also control CETP and hepatic lipase activity. Therefore, HDL may also increase in hypothyroidism, but atherogenic lipids increase are proportionally more and an atherogenic microenvironment occurs.^8^

In our study, both the patient group and the control group consisted of euthyroid patients. So thyroid dysfunction should not be the cause in this dyslipidemia profile. However, it is still a question of whether the dyslipidemic picture is due to the statistically significant high TSH level in the patient group compared to the control group. There are studies showing increased dyslipidemia with increased TSH level even in the normal range.^14^,^15^ In the HUNT study, one of the most important of these, even in the normal ranges of TSH, increases in LDL, TC and non-HDL levels were found with the increasing level of TSH.^16^ However, this relationship was not detected in the study conducted in by Topaloglu et al.; there was no significant correlation between lipid parameters and TSH in euthyroid patients.^17^

Thyroid autoimmunity may contribute to the pathogenesis of dyslipidemia. Cellular immunity defects are known to be the major factor in the pathogenesis of autoimmune thyroiditis. T helper cells, which cannot be suppressed due to suppressor T cell function defect, secrete many mediators such as IFN-ɤ, IL-2 and TNF-α, causing their increase in the circulation.^7^ These mediators were found to be associated with lipogenesis in a later animal study.^18^

There are many studies that shows the relationship between thyroid autoimmunity and hyperlipidemia without thyroid dysfunction such as our study. In the PORMETS study conducted in Portugal, there was a relationship between Anti-TPO positivity and metabolic syndrome components, especially TG levels (OR: 0.321; 95% CI 0.124-0.836).^19^ In a study conducted in China ;a significant relationship was found between Anti-TPO and Anti-Tg positivity and hyperlipidemia.^4^ In an another Chinese study ;TC and HDL levels were found higher in patients with Anti-TPO positivity in middle-aged euthyroid patients.^20^ In the studies by Tamer et al and Mazaheri et al Anti-TPO was negatively correlated with HDL cholesterol levels, independent of thyroid function.^21^,^22^ In the study of Bıyıklı et al.; LDL and TG levels were found to be significantly high in euthyroid hashimoto patients.^23^ In another study conducted at our University, a significant relationship was noted between thyroid autoimmunity and TC and LDL cholesterol in pubertal girls (p <0.01).^24^

**Table-III:** Correlation analysis results between thyroid autoantibodies and lipid panel.

	Anti-TPO		Anti-TG	

	r value	p value	r value	p value
Low-density lipoprotein (LDL)	0.331	<0.001	0.318	0.001
High-density lipoprotein (HDL)	0.149	0.121	0.142	0.138
Triglycerides	0.267	0.005	0.218	0.022
Total cholesterol	0.316	0.001	0.301	0.001

In our study, there was a significant correlation between Anti-TPO and Anti-TG level and TC, LDL and TG, but HDL was not correlated with thyroid autoantibodies. When the studies are examined that giving correlation analysis; Chen et al. Found higher TC, LDL and TG and lower HDL levels in Anti-TPO and Anti-TG positive patients, but only TG level correlated significantly with antibodies.^4^ According to the two study conducted in Turkey first by Topaloglu et al. Total cholesterol and LDL cholesterol were positive and HDL cholesterol was negative correlated with Anti-TPO and Anti-Tg antibodies.^17^ Second study conducted by Tamer et al.;TG levels were positive, HDL levels were negative correlated with Anti-TPO levels and TG and non HDL cholesterol levels were found to be significantly positive correlated with Anti-TG antibody as in our study.^21^

While the atherogenic lipid changes in overt and subclinical hypothyroidism and their adverse cardiovascular effects have now been well studied, there are few studies on the relationship between thyroid autoimmunity and hyperlipidemia. In our study, we clearly demonstrated the effect of thyroid autoimmunity on atherogenic lipid profile which is independent of thyroid dysfunction. In this respect, we think that our study has an important contribution to the literature. Our study’s results reinforce the data of previous studies on this subject. However, it’s still a matter of debate whether or not early intervention is required to prevent hyperlipidemia and related vascular complications, or whether it should be followed differently than the normal population even in euthyroid phase in patients with autoimmune thyroiditis. Large randomized prospective studies are needed to reach more precise conclusions.

### Limitations of the study

Our study is a single-center study with a relatively low number of patients. More atherogenic lipid subtypes such as Lipoprotein a (Lp(a)) have not been studied. In addition, no comparison was made with subclinical and overt hypothyroid patient groups as control group. It was not investigated whether the adverse lipid profile of the patients was related to Anti-TPO and Anti-Tg titers. Long-term cardiovascular results of this adverse lipid profile in patients were not followed.

## CONCLUSION

In this study, we found that TC, LDL, TG and HDL levels were significantly higher in patients with autoimmune thyroiditis even in euthyroid phase. When correlation analysis was made with autoantibodies, TC, TG and LDL cholesterol levels were significantly positive correlated with Anti-TPO and Anti-Tg. Our current study is important in that we can demonstrate the dyslipidemic effect of thyroid autoimmunity in our patient group. Larger randomized controlled studies are needed to clarify the dyslipidemia and atherogenic effects of thyroid autoimmunity.

### Authors’ Contribution:

**HC:** Conceived the study, enrolled patients, is responsible for integrity of research.

**TD:** Statistical analysis and the last controls.

**CV:** Prepared the manuscript

**AT:** He translated the article to the English and prepared the tables.
